# Risk factors for recurrent obstetric anal sphincter injury (rOASI): a systematic review and meta-analysis

**DOI:** 10.1007/s00192-015-2893-4

**Published:** 2015-12-16

**Authors:** Swati Jha, Victoria Parker

**Affiliations:** Department of Urogynaecology, Sheffield Teaching Hospitals, Level 4 Jessop Wing, Tree Root Walk, Sheffield, S10 2SF UK

**Keywords:** OASI, Anal sphincter injury, Recurrence, Subsequent delivery, Third/fourth-degree tear

## Abstract

**Objectives:**

The objective of this study was to estimate the risk of recurrent obstetric anal sphincter injury (rOASI) in women who have suffered anal sphincter injury in their previous pregnancy and analyse risk factors for recurrence through a systematic review and meta-analysis.

**Data sources:**

A review was performed according to Preferred Reporting Items for Systematic Reviews and Meta-Analyses (PRISMA) guidelines. Searches were made in Ovid MEDLINE (1996 to May 2015), PubMed, EMBASE and Google Scholar, including bibliographies and conference proceedings.

**Methods of study selection:**

Observational studies (cohort/case–control) evaluating rOASI and risk factors were selected by two reviewers who also analysed methodological quality of those studies. Pooled odds ratios (OR) for rOASI and individual risk factors were calculated using RevMan 5.3.

**Tabulation, integration and results:**

From the eight studies assessed, overall risk of rOASI was 6.3 % compared with a 5.7 % risk of OASI in the first pregnancy. The risk in parous women with no previous OASI was 1.5 %. Factors that increased the risk in a future pregnancy were instrumental delivery with forceps [OR 3.12, 95 % confidence interval (CI) 2.42–4.01) or ventouse (OR 2.44, 95 % CI 1.83–3.25), previous fourth-degree tear (OR 1.7, 95 % CI 1.24–2.36) and birth weight ≥4 kg (OR 2.29, 95 % CI 2.06–2.54). Maternal age ≥35 years marginally increased the risk (OR 1.16, 95 % CI 1–1.35).

**Conclusion:**

The overall rate of rOASI and associated risk factors for recurrence are similar to the rate and risk factors of primary OASI. Antenatal decisions could be based on assessment of foetal weight and intrapartum decisions based upon the requirement for an instrumental delivery.

**Electronic supplementary material:**

The online version of this article (doi:10.1007/s00192-015-2893-4) contains supplementary material, which is available to authorized users.

## Introduction

The incidence of obstetric anal sphincter injury (OASI) appears to be rising, with rates reported between 0.6 % in Finland [1] and 19.3 % in a primiparous population in the USA [2]. A recent UK survey showed rates ranging from 0 to 8 %, with a median of 2.85 % [3], which is an increase from the previous reported rates of 1 % [4]. Furthermore, another UK survey reported a trebling in the incidence of OASI from 2000 to 2012 [5]. Similar increasing rates of OASI have been reported from Australia [6], Scandinavia [1] and the USA [7]. Aside from the usual concerns with OASI related to faecal incontinence, perineal pain, dyspareunia, psychological problems, such as depression, and overall impact on quality of life, concerns about recurrence can deter women from having another vaginal delivery [8] or even from futher childbirth. Reported rates of recurrent OASI (rOASI) are variable, ranging from 2 % [9] to 13.4 % [10]; risk factors are poorly reported. A better understanding of the overall risk of recurrence and factors that contribute to that risk would enable women and caregivers to make better informed decisions with regards future childbearing options and mode of delivery.

The aim of this study was to systematically estimate the risk of rOASI in women who had an anal sphincter injury in a previous pregnancy and to analyse risk factors for recurrence through a systematic review and meta-analysis.

## Materials and methods

### Eligibility criteria, information sources and search strategy

**Fig. 1:**
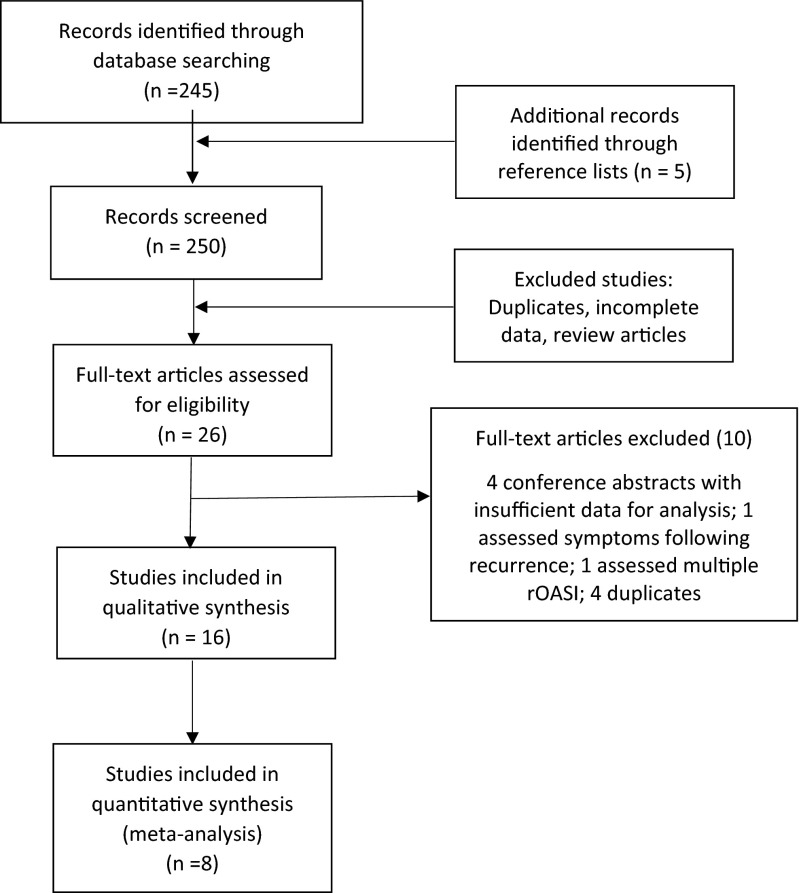
Study search results

Ovid MEDLINE (1996 to May 2015), PubMed, EMBASE and Google Scholar were searched using the terms OASI, anal sphincter injury, recurrence and subsequent delivery, with no language restrictions. One study in French [11] was identified initially but subsequently found to be unsuitable as it included third and fourth subsequent OASI events, which was different to the remaining studies, which assessed the risk of a second OASI. Reports from reference lists of identified studies were retrieved. All databases were searched up to 20 May, 2015. A manual search of reference lists of identified articles and conference proceedings of major national and international meetings was also conducted. Investigators involved in the field were contacted to locate unpublished data. A protocol was developed with explicitly defined objectives, criteria for selection and quality assessment of studies, primary and secondary outcomes and statistical methods. Meta-analysis of Observational Studies in Epidemiology (MOOSE) guidelines for reporting meta-analyses of observational studies were followed [12] (Fig. 1).

### Study selection

Observational studies (cohort or case–control) analysing rOASI and risk factors were selected. Where data were duplicated between articles, the most recent article or that with the largest sample size was used. This resulted in the exclusion of four studies [13–16]. Where data were incomplete, authors were contacted to obtain data and a reminder was sent 2 weeks later.

### Assessment of risk of bias

Study methodological quality was assessed using six of the 14 items adapted from the Quality Assessment Tool for Observational Cohort and Cross-Sectional Studies developed by the National Institutes of Health (NIH), US Department of Health and Human Services [17]. The remaining items were not of significance for this review and were excluded. Each item was scored as either yes, no or unclear. The six NIH assessment criteria were as follows:

1. Was the study population clearly specified and defined? When the authors described the group of people from which the study participants were selected or recruited, using demographics, location, and time period, the response was yes. When this was not defined, it was no.
2. Were all participants selected or recruited from the same or similar populations (including the same time period)? When the cohort of rOASI was from the same population defined above, the answer was yes, but when patients with rOASI included patients who had delivered outside of the initial population, the response was no.
3. Was the sample size adequate? We selected observational cohort studies, and they did not report on power or sample sizes because analyses are exploratory in nature. Authors of this review agreed that an adequate sample size would be a study population >100, with previous OASI and having another vaginal delivery.
4. Was the timeframe sufficient so that one could reasonably expect to see an association between exposure and outcome if it existed? It was agreed that women followed up for a 10-year period from their first delivery was a sufficient timeframe. The reason was that most women who contemplate a second pregnancy will do so within 10 years of the index pregnancy.
5. Were the outcome measures (dependent variables) clearly defined? The reference standard used to define OASI was according to Sultan’s classifications [4]. Where this definition was referenced, we qualified the study as clearly identifying the outcome measure. Where no reference was documented and only the term third-/fourth-degree tear was used, the study qualified as not defining outcome. Where other methods such as database recognition were used, the study was deemed unclear in defining outcomes.
6. Were key potential confounding variables measured and adjusted statistically for their impact on the relationship between exposure(s) and outcome(s)? Where risk factors were analysed in women with previous OASI so that odds ratios (OR) and confidence interval (CI) calculations were feasible, we identified the study as measuring variables impacting on exposure and outcome.

**Fig. 2:**
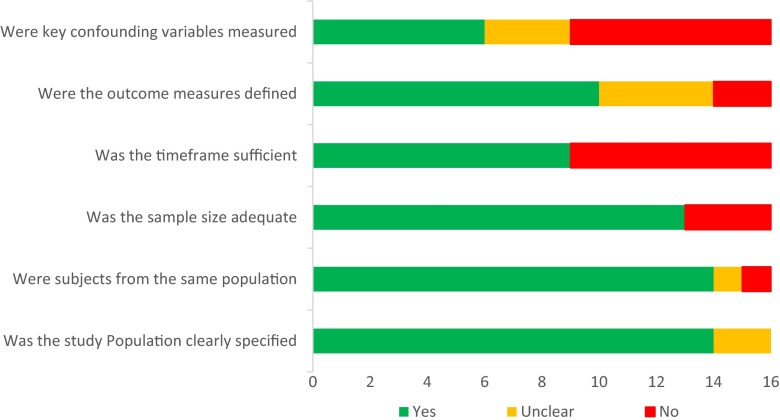
Methodological quality of studies in this systematic review

Studies that did not score in any category were excluded from the analysis; remaining studies were categorised as high or low quality on this basis. Where there was insufficient information, the study was scored as unclear. Both authors (SJ and VP) independently assessed study quality, and disagreements were resolved by consensus. Quality assessment of all studies in the review is shown in Fig. 2.

### Data extraction and synthesis

The authors independently extracted data from each eligible study using a standardised data abstraction form. For each study, data were extracted on the risk of OASI in the index pregnancy and risk of recurrence (rOASI) in a subsequent pregnancy. Where available, we also obtained data regarding OASI in the second pregnancy occurring for the first time following a previous uncomplicated vaginal delivery. Reported ORs and CIs for recurrence were used and calculated when adequate data were available. Where direct calculations (insufficient data or not reported) were not possible, percentages were stated. Where articles presented adjusted ORs, these were entered into the primary analysis. Where this was not available, crude ORs were used. Weighting of studies was assigned according to the inverse of variance. A fixed-effects model was undertaken in the absence of heterogeneity and a random effects model when this was >50 %. Heterogeneity was calculated using the Cochrane Q test and quantified with the I^2^ statistic; I^2^ > 50 % was considered significant heterogeneity.

None of the cohort studies could be analysed for meta-analysis because they did not compare outcomes between patients with and without OASI in a previous pregnancy. Where data for ORs were provided, these were analysed separately, but results could not be pooled for the meta-analysis. For case–control studies, where data were provided for risk factors in subsequent vaginal deliveries with a rOASI compared with those who did not, results could be pooled to conduct a meta-analysis. One study [9] reported continuous data using means and standard deviations (SD) for representing risk factors; however, we were unable to use this information, as it could not be combined with data from other studies. Two studies [8, 9] compared outcomes in the second pregnancies in women with and without a previous OASI in their first pregnancy.

RevMan 5.3 was used to calculate OR and 95 % CIs for all risk factors where more than one study reported on that specific risk. Data were obtained for all reported risk factors, including episiotomy, forceps, ventouse, grade of previous tear (third- or fourth-degree), induction, augmentation, epidural, birthweight (BW), maternal age, interval between pregnancies and Asian ethnicity. Risk factors such as shoulder dystocia, two previous OASI, infant’s sex and head circumference and maternal body mass index (BMI) were each reported by only one study, so a pooled OR calculation was not possible.

## Results

### Study selection

All studies identified as being suitable were in English. We reviewed 250 abstracts and 26 full-text articles: 16 were used for qualitative assessment, and of them, eight formed the basis of the meta-analysis. Three studies were conference abstracts, and the remaining 13 were full-length articles (Table 1). Sample size was number of patients in the study who had a known OASI in a previous pregnancy and proceeded to have another vaginal delivery. Sample size ranged from 53 to 43,583. Based on inclusion criteria, the number of patients in this review was 99,042, with an overall average rate of rOASI being 6.3 %. The risk of OASI in the first pregnancy was 5.7 % and in a parous woman who had no prior OASI was 1.5 %.

**Table 1:** Study overview

Study year	Authors (location)	Study period	Sample size (rOASI)	Type of study	Risk factors assessed	Risk of OASI in 1st pregnancy (%)	Risk of 1st OASI in 2nd pregnancy (%)	Risk of recurrence (%) (OR; CI)
1999	Payne et al. (US) [23]	1990–1994	178 (19)	Cohort	--	10.2	3.6	10.7 (3.4; 1.8–6.4)
1999	Peleg et al. (US) [2]	1978–1995	774 (58)	Case–control	Episiotomy	19.3	3.2	7.5 (2.5; 1.8–3.4)
2003	Harkin et al. (Ireland) [22]	1997–1999	45 (2)	Cohort	--	1.7	0.8	4.4 (−−)
2004	Elfaghi et al. (Sweden) [20]	1973–1997	10,807 (478)	Cohort	--	1.3	0.8	4.4 (5.98; 5.44–6.58)
2005	Dandolu et al. (US) [26]	1990–2001	14,990 (864)	Case–control	Forceps; ventouse, episiotomy; grade of previous tear	7.31	--	5.76 (0.78; 0.72–0.83)
2009	Burton et al. (UK) [21]	2001–2008	53 (2)	Cohort	--	4	--	3.8
2012	Jango et al. (Denmark) [27]	1997–2010	7336 (521)	Case–control	Forceps; ventouse; episiotomy; induction; epidural; presentation; birthweight; head circumference; age; grade of OASI in first; shoulder dystocia	4.6	--	7.1 (5.91; 6.5–7.7)
2012	Baghestan et al. (Norway) [30]	1967–2004	13,305 (750)	Case–control	Forceps; ventouse; birthweight; age;	2.8	0.8	5.6 (4.2; 3.9–4.5)
2012	Parmar et al. (US) [7]	1991–2004	43,583 (2648)	Case–control	Forceps; ventouse; birthweight; age	11.6	1.4	6.1 (3.79; 3.60–3.98)
2013	Basham et al. (US) [25]	2005–2010	685 (22)	Case–control	Forceps; ventouse; episiotomy; grade of OASI in 1st	--	--	3.2 (−−)
2014	Yogev et al. (Israel) [9]	2000–2012	166 (4)	Case–control	Forceps; ventouse; grade of OASI in 1st	0.6	0.3	2 (2.3–18.3)
2014	Doumouchtsis et al. (UK) [19]	2001–2013	307 (28)	Case–control	Head circumference; Birth wt; Age; Ethnicity; smoking; mode of delivery	--	--	9.12 (−−)
2014	Boggs et al. (Canada) [18]	2006–2010	1923 (102)	Case–control	Episiotomy; augmentation; induction; instrumental	5.3	--	5.3 (1; 0.8–1.2)
2014	Ali et al. (Ireland) [10]	2010–2012	82 (11)	Case–control	Episiotomy; forceps; ventouse	3.4	1	13.4
2014	Edozien et al. UK[8]	2004–2012	17,352 (1249)	Cohort	Episiotomy; forceps; ventouse; grade of tear; birthweight; age; shoulder dystocia	3.8	1.3	7.2
2015	Ampt et al. (Australia) [24]	2001–2011	4808 (276)	Case–control	Episiotomy; ethnicity; induction; instrumental; birthweight; epidural; age	4.5	--	5.7 (−−)

*rOASI* recurrent obstetric anal sphincter injury, *OR* odds ratio, *CI* confidence interval

### Study characteristics

All studies demonstrated an increase in rOASI risk except that of Dandolu et al. [26, which showed a decrease (OR 0.78, CI 0.72–0.83). One study [18] compared risk factors for primary OASI to rOASI and found no difference (OR 1, 95 % CI 0.8–1.2). Several studies presented just ORs and CIs, but in the absence of raw numbers, their data could not be pooled with the other studies, in which dichotomous data were provided, and were excluded from the analysis [7–9, 18–20]. Of the 16 studies, five were cohort studies [8, 20–23] and the remaining were case–control. Three studies had <100 participants. Cohort studies did not permit relative risk (RR) or OR calculations.

### Risk of bias

Studies varied in methodological quality, with six (37.5 %) fulfilling all six criteria and six (37.5 %) failing to meet at least three criteria (Fig. 2). As expected, there was evidence of significant heterogeneity in the case–control studies. Heterogeneity was significant for follow-up duration, and confounding variables were not routinely recorded. Funnel plots for risk factors when more than three studies were included were symmetrical for forceps, ventouse, tear grade and maternal age.

### Synthesis of results

A summary of results is shown in Table 2.

**Table 2:** Published and calculated relative risk (RR) and odds ratios (OR) for case–control studies

Risk factor		No. studies	No. women	No. exposures to risk factor	RR (95 % CI)	I^2^ (%)	Pooled OR (95 % CI)
Episiotomy		7 [2, 10, 18, 24–27]	30,588	10,504	1.15 (0.90–1.47)	89	1.26 (0.69–2.28)
Forceps		5 [10, 25–27, 30]	36,398	488	3 (2.36–3.81)	24	3.12 (2.42–4.01)
Ventouse		5 [10, 25–27, 30]	36,398	1501	2.32 (1.74–3.08)	51	2.44 (1.83–3.25)
Grade of tear		4 [9, 25–27]	23,267	5984	1.4 (1.3–1.5)	77	1.7 (1.24–2.36)
Induction		2 [24, 27]	12,144	3507	1.08 (0.84–1.38)	77	1.09 (0.8–1.50)
Epidural		2 [24, 27]	12,144	1432	0.98 (0.78–1.24)	25	0.97 (0.77–1.23)
Birth weight	3–3.5 kg	2 [27, 30]	20,641	4929	0.55 (0.48–0.64)	0	0.48 (0.41–0.57)
	3.5–4 kg	2 [27, 30]	20,641	8295	0.88 (0.82–0.95)	26	0.81 (0.72–0.91)
	>4 kg	4 [24, 25, 27, 30]	26,134	7064	1.69 (1.6–1.79)	0	2.29 (2.06–2.54)
	>4.5 kg	2 [27, 30]	20,641	1342	2.59 (2.25–2.99)	0	2.89 (2.45–3.40)
Maternal age > 35		3 [24, 27, 30]	25776	3164	1.14 (1–1.29)	0	1.16 (1–1.35)
Asian ethnicity		2 [24, 25]	5493	993	0.84 (0.64–1.09)	0	0.81 ( 0.58–1.11)

#### Episiotomy

It was not possible to perform this analysis separately, as sufficient data were not provided in the various studies. Eight [2, 10, 18, 21, 24–27] studies assessed the effect of episiotomy on rOASI. Of these, four did not state the type of episiotomy, one [25] included both midline and mediolateral episiotomy and the remaining three [21, 24, 27] had exclusive mediolateral episiotomies. Midline episiotomies have a higher risk of OASI [28, 29]. Edozien et al. reported a decrease in the risk of rOASI when a mediolateral episiotomy was performed [8].

#### Forceps

There was a significant increase in the risk of rOASI following forceps delivery (OR 3.12; CI 2.42–4.01). Five studies assessed the impact of a forceps delivery on rOASI [10, 25–27, 30] . A further study by Yogev et al. [9] also showed that forceps significantly increased the risk of rOASI (OR 20, 95 % CI 6.6–60.3); however, that study could not be used for the meta-analysis because raw data were not available. Included studies did not differentiate between the type of forceps, i.e. rotational versus midcavity or low-outlet forceps.

#### Ventouse

There was a significant increase in the risk of rOASI with at least a doubling of incidences. Five studies reported on the impact of a ventouse delivery on rOASI [10, 25–27, 30] but did not differentiate between the different types of cups, i.e. kiwi, silicon or metal. They also failed to differentiate between rotational ventouse and ventouse extraction. One study looked at sequential use of ventouse and forceps.

#### Grade of previous tear (third- or fourth-degree)

A previous fourth-degree tear increased the odds of rOASI (OR 1.7, 95 % CI 1.24–2.36) [9, 25–27]. One study failed to show an association [25] but did report on increasing caesarean section (CS) rates for women with a previous fourth-degree tear. In addition, the study had no pregnancy data on more than half of all women who had a primary OASI.

#### Birthweight

Increasing BW >4 kg was associated with an increase in rOASI rates [BW 4 kg, (OR 2.29, 95 % CI 2.06–2.54); BW 4.5 kg (OR 2.89, 95 % CI 2.45–3.40)] [24, 25, 27, 30]. BW >5 kg was even more significant, with an OR 9.92, 95 % CI 7.44–13.22 reported by Parmar et al. [7] and an OR 4.5, 95 % CI 2.8–6.99 reported in another study [30]. Two studies [27, 30] document decreasing rOASI rates when BW is <4 kg. One study [25] reported on the difference in BW between the primary OASI and rOASI pregnancies.

#### Time between pregnancies

Five studies [8, 19, 24, 27, 30] report on interpregnancy interval and association with rOASI. No studies reported on a positive association, with 95 % CI crossing unity in all studies.

#### Maternal age

Two studies examined maternal age >40 years for rOASI, and both demonstrated an increased risk, with OR of 1.34 (95 % CI 1.14–1.58) [7] and 1.95 (95 % CI 1.06–3.55) [30]. Three studies [6, 27, 30] analysed rOASI in women >35 years and demonstrated a slight increase in risk (OR 1.16, 95 % CI 1–1.35). Baghestan et al. showed no association of rOASI in patients between 35 and 40 years but did show an association in those >40s [30. This suggests increasing age is a risk factor for rOASI. Ali et al. [10], however, failed to show an association between age and risk of recurrence, but numbers in that study were small.

#### Asian ethnicity

Two studies reported on Asian ethnicity [24, 25] as an underlying risk factor for rOASI. This failed to reach statistical significance (OR 0.81, 95 % CI 0.58–1.11).

#### Induction

Three studies assessed induction of labour in the rOASI group [18, 24, 27]. Two studies could be combined and showed no significant association (OR 1.09, 95 % CI 0.8–1.50). The third study could not be used for analysis, but results were similar [18].

#### Epidural

Two studies [24, 27] reported on the effect of epidural analgaesia and failed to show an association with rOASI (OR 0.86; 95 % CI 0.62–1.18).

#### Two Previous OASI

One study [30] assessed the risk of OASI in third pregnancies. After two previous OASIs, this was particularly high (absolute risk 9.55, adjusted OR 10.6). Women who did not have OASI in the first but did in the second delivery were nine times more likely to have one in the third pregnancy. Women who had an OASI in the first but not the second pregnancy were still at a marginally increased risk in the third (3.1 %).

#### Shoulder dystocia

Two studies reported on shoulder dystocia as a risk factor, and both documented a significant increase in rOASI risk. Jango et al. [27] reported an OR of 3.7, 95 % CI 2.2–6.4, whereas Edozien et al. [8] reported an even higher risk (OR 4.27, 95 % CI 3.83–4.76).

#### Sex of the infant

One study reported on the sex of the child in the subsequent pregnancy and found no association with rOASI risk (OR 1.12, 95 % CI 0.87–1.44).

#### Head circumference

Jango et al. [27] reported an association between head circumference, BW and rOASI. For a fixed BW, a larger head circumference was associated with a lower risk of rOASI.

#### Maternal BMI

Jango et al. [27] reported on the impact of maternal BMI and rOASI. The association was nonsignificant, with OR 1.02 and 95 % CI 1.00–1.04).

#### Occipit posterior position

One study [27] reported on occipitoposterior position of the baby and the risk of rOASI, showing a significant association, with OR 1.73, 95 % CI 1.14–2.63).

#### Labour augmentation

One study [18] reported no association between augmentation and rOASI, whereas another showed a positive association (OR 1.5, 95 % CI 1.14–1.97) [27].

## Discussion

In this systematic review, we found that an instrumental delivery with either forceps or ventouse, BW >4 kg, shoulder dystocia or a prior fourth-degree tear all increase the risk of rOASI in a future pregnancy. Maternal age >35 years appears to marginally increase the risk. The overall risk of recurrence was 6.3 %, with population-based studies demonstrating a clinically nonsignificant increase in OASI rates (from 5.7 %) in subsequent pregnancies when risk factors stayed the same.

The strength of this systematic review is the large number of patients and the fact that population-based cohort studies from Australia, Scandinavia, Ireland, UK and the USA form part of the study. This was a very diverse population group, and the studies included were testing the same set of risk factors. Population studies that form part of the review relied on diagnostic coding from databases, which are consistently accurate [31–33]. However, they do have limitations, and it is impossible to be 100 % certain that women who had an OASI in the index pregnancy were primiparous, particularly if they delivered in another country.

The main weakness of the systematic review is that the included studies reported individual risk factors in a subsequent pregnancy but little about the association between them. Factors rarely occur in isolation, e.g. forceps delivery and a baby >4 kg or a large baby and shoulder dystocia usually occur in conjunction. Studies [27] show that patients with rOASI usually have multiple risk factors, with almost half of all women with rOASI having more than one risk factor. It is difficult to determine if the effect of these individual risk factors is cumulative or compounded. It was not possible to control for some confounding factors, such as experience of the accoucheur, the angle and type of episiotomy or foetal head circumference. In addition, our systematic review did not allow for changing demographic data following the index pregnancy in the population being studied. These include changing body mass index (BMI) and changing medical morbidity, in particular. It is also possible that where follow-up was not long enough, not all women with a subsequent vaginal delivery following an OASI would have been included in the studies that formed part of this review.

Here we report a marginally increased risk of rOASI in women who had a fourth-degree tear in their index pregnancy, but those women were more likely to undergo a CS [8, 9, 25] in subsequent pregnancies. This would result in inaccurate reporting of the actual risk of recurrence and bias in interpretation of the actual OR for this risk. It is also possible that women delayed childbearing beyond the time period included in the studies. Although we used 10 years as a cutoff for good-quality studies, it is possible some women were missed because they delayed further childbearing beyond 10 years. This is particularly true given that women are now delaying childbearing to a later age. None of the studies in the review commented on underlying symptoms or endoanal physiology as a guide for mode of delivery in future pregnancies. This would be a major confounding factor when analysing results, as it would introduce further heterogeneity.

Some risk factors were not encountered in the studies reported and hence could not be commented on. These include accoucher experience, baby position during delivery and length of the second stage of labour. Studies appear to consistently show that the first vaginal delivery is at substantial risk for OASI, with subsequent vaginal delivery being at an even greater risk. A systematic review analysing risk factors for OASI in the first delivery [34] found they were similar for rOASI. Population-based studies in this review consistently showed an increase in the rate of rOASI. This increase ranged from 4.6 % to 7.1 % in Denmark [27], 2.8 % to 5.6 % in Norway [30], 1.3 % to 4.4 % in Sweden [20], 3.4 % to 13.4 % in Ireland [10], 4.5 % to 5.7 % in Australia [6] and 3.8 % to 7.2 % in the UK [8]. The study by Dandolu et al. from the USA [26] was the only one that showed a decrease in rOASI risk compared with the first OASI (7.31 vs 5.76 %), but this was linked to a decrease in the number of forceps deliveries performed at the time of the index pregnancy compared with the first delivery. Similarly, Boggs et al. [18], reporting on a population-based cohort from Canada, showed no change in the rate of rOASI compared with the first (5.3 % vs 5.3 %). This could be related to the fact that their study was conducted over a short (4-year) period. Women with previous OASI tend to delay subsequent childbearing, and hence, that study may not have captured the full range of women with OASI who went on to have subsequent rOASI. Two further US-based studies [2, 7] showed an overall reduction in the percentage of women with an rOASI, but the ORs and CIs demonstrated an increased risk of rOASI, which is probably linked to a decrease in exposure to risk factors over the time period studied. In addition, both those studies were relatively old, dating back to the 1970s and 1990s.

Our systematic review found that women who had an OASI in the first pregnancy were more likely to have a CS in the subsequent pregnancy or abstain from subsequent pregnancy [8, 27, 30], This has been reported in other studies [16, 35]. This is partly influenced by the clinician women see in a subsequent pregnancy, and studies showing that 22 % of obstetricians in the UK [36] would recommend an elective CS to prevent faecal incontinence in a future pregnancy. This has implications for clinical practice and is a major cause for the increasing CS rates. Careful counselling of women with OASI in a previous pregnancy is required so they can make a better-informed decision regarding avoiding an rOASI. The only risk factor women and their doctors are aware of prior to the subsequent pregnancy is maternal age and a previous fourth-degree tear. BW >4 kg may be detected antenatally; however, this method is marred by inaccuracies in accurately estimating foetal BW. Other risk factors, including forceps or ventouse delivery, occipitoposterior foetal position and shoulder dystocia develop intrapartum, when very little can be done to prevent them. Awareness of their risks of causing rOASI may influence decisions regarding mode of delivery.

Women should be informed antenatally of factors that increase the risk of rOASI in their subsequent pregnancy. Some of these risk factors can be identified before labour, including estimated foetal weight, previous fourth-degree tear and maternal age; others may occur during labour. Women’s choices on mode of delivery should be recorded to aid decision making during labour. The only means of eliminating the risk of rOASI is an elective CS delivery. However, following OASI in the index pregnancy, a CS has attendant risks of increased morbidity when compared with a vaginal delivery: 11.3 % compared with 4.2 % following a vaginal delivery (RR 2.7, 95 % CI 2.6-2.8) [37]; 2.3 CS deliveries were required to prevent one case of anal incontinence.

Interventions aimed at reducing OASI, such as manually supporting the perineum during childbirth [38–41], have been poorly studied, and further studies are required to assess the impact of these manoeuvres on rOASI. Given the benefits of OASI reduction identified by perineal protection [39, 40] in the first pregnancy, this may be considered in women opting for a vaginal delivery in order to prevent rOASI until more robust evidence is available.

## Electronic supplementary material

Below is the link to the electronic supplementary material.

ESM 1 (DOC 59 kb)
