# Supplementary material for: Risk factors for recurrent obstetric anal sphincter injury (rOASI): a systematic review and meta-analysis
Source: Int Urogynecol J. 2015 Dec 16;27:849–57. doi: 10.1007/s00192-015-2893-4 (PMC4879153; doi:10.1007/s00192-015-2893-4)
Supplement: Supplementary file 1 — (DOC 59 kb) [file 192_2015_2893_MOESM1_ESM.doc]

**Reporting Checklist for Meta-analyses of Observational Studies (MOOSE) for PLoS Medicine**

|  | Reported? | MS page |
| --- | --- | --- |
| Reporting of background should include: |  |  |
| Problem definition | Yes | 5 |
| Hypothesis statement | Yes | 5 |
| Description of study outcome(s) | Yes | 6 |
| Type of exposure or intervention used | Yes | 7 |
| Type of study designs used | Yes | 7 |
| Study population | Yes | 7 |
| Reporting of search strategy should include: |  |  |
| Qualifications of searchers (eg, librarians and investigators) | Yes | 1 |
| Search strategy, including time period included in the synthesis and keywords | Yes | 7 |
| Effort to include all available studies, including contact with authors | Yes | 7 |
| Databases and registries searched | Yes | 7 |
| Search software used, name and version, including special features used (eg, explosion) | Yes | 7 |
| Use of hand searching (eg, reference lists of obtained articles) | Yes | 7 |
| List of citations located and those excluded, including justification | Yes |  |
| Method of addressing articles published in languages other than English | Yes | 7 |
| Method of handling abstracts and unpublished studies | Yes | 7 |
| Description of any contact with authors | Yes | 7 |
| Reporting of methods should include: |  |  |
| Description of relevance or appropriateness of studies assembled for assessing the hypothesis to be tested | Yes | 12 |
| Rationale for the selection and coding of data (eg, sound clinical principles or convenience) | Yes | 12 |
| Documentation of how data were classified and coded (eg, multiple raters, blinding, and interrater reliability) | Yes | 8,9 |
| Assessment of confounding (eg, comparability of cases and controls in studies where appropriate) | Yes | 9,18 |
| Assessment of study quality, including blinding of quality assessors; stratification or regression on possible predictors of study results | Yes | 8,9 |
| Assessment of heterogeneity | Yes | 10 |
| Description of statistical methods (eg, complete description of fixed or random effects models, justification of whether the chosen models account for predictors of study results, dose-response models, or cumulative meta-analysis) in sufficient detail to be replicated | Yes | 10 |
| Provision of appropriate tables and graphics | Yes | Attached Tables and Figures |
| Reporting of results should include: |  |  |
| Graphic summarizing individual study estimates and overall estimate | Yes | Table 2 |
| Table giving descriptive information for each study included | Yes | Table 1 |
| Results of sensitivity testing ( eg, subgroup analysis) | No | NA |
| Indication of statistical uncertainty of findings | Yes | 17,18 |
| Reporting of discussion should include: |  |  |
| Quantitative assessment of bias (eg, publication bias) | Yes | 19 |
| Justification of exclusion (eg, exclusion of non-English-language citations) | Yes | 9,10 |
| Assessment of quality of included studies | Yes | Figure 2 |
| Reporting of conclusions should include: |  |  |
| Consideration of alternative explanations for observed results | Yes | 18,19 |
| Generalization of the conclusions (ie, appropriate for the data presented and within the domain of the literature review) | Yes | 17 |
| Guidelines for future research | Yes | 21 |
| Disclosure of funding source | Yes | 1 |

- Stroup DF, Berlin JA, Morton SC, Olkin I, Williamson GD, Rennie D, Moher D, Becker BJ, Sipe TA, Thacker SB. Meta-analysis of observational studies in epidemiology: a proposal for reporting. Meta-analysis Of Observational Studies in Epidemiology (MOOSE) group. JAMA 2000; 283(15):2008-2012.
